# Synergistic Effects of Fertilization and Reclamation Age on Inorganic Phosphorus Fractions and the *pqqC*-Harboring Bacterial Community in Reclaimed Coal Mining Soils

**DOI:** 10.3390/microorganisms13122855

**Published:** 2025-12-16

**Authors:** Zhiwen Fang, Kunli Liu, Yunlong Jiang, Jianfang Wang, Zhuomin Song, Huisheng Meng, Xianjun Hao, Jie Zhang, Xiangying Wang

**Affiliations:** 1 College of Life Science, Shanxi Agricultural University, Jinzhong 030801, China; fzw399555@163.com (Z.F.); lkli121@163.com (K.L.); whoissssssszm725@163.com (Z.S.); 2 College of Resources and Environment, Shanxi Agricultural University, Key Laboratory of Sustainable Dryland Agriculture of Shanxi Province, Taiyuan 030031, China; 17339511026@163.com (Y.J.); 13453845190@163.com (J.W.); huishengmeng@126.com (H.M.); haoxianjun660@126.com (X.H.); zhangjzh@sxau.edu.cn (J.Z.)

**Keywords:** reclaimed soil, inorganic phosphorus fractions, *pqqC* gene, organic manure, chemical fertilizer

## Abstract

Fertilization is an effective measure to rapidly improve soil quality in reclaimed mining areas. However, the combined effects of fertilization regimes and reclamation age on phosphorus (P) fraction transformation and the *pqqC*-harboring microbial community in reclaimed soils remain unclear. In this study, we investigated the dynamics of inorganic P fractions and the *pqqC*-harboring bacterial community under different fertilization treatments (no fertilizer: CK; chemical fertilizer: CF; organic manure: M) and reclamation ages (1, 5, and 10 years) in a coal mining reclamation area of Shanxi Province, using long-term field experiments combined with high-throughput sequencing. Results showed that compared with the CF and CK treatments, the M treatment significantly increased soil organic matter (SOM), available P (AP), and total nitrogen (TN) content, and promoted the conversion of moderately labile P (NaOH-Pi) to labile P fractions (H_2_O-Pi, NaHCO_3_-Pi). Meanwhile, the *pqqC* gene abundance increased with reclamation age, with the M treatment maintaining the highest levels in all fertilization regimes. Co-occurrence network analysis of core species revealed that the number of connections gradually decreased and the network structure simplified with increasing reclamation age. Correspondingly, the microbial community transitioned from an initial stage characterized by rapid response and intense competition to a stable phase. Specifically, *Pseudomonas* spp. played a key role in P mobilization. Structural equation modeling (SEM) further demonstrated that reclamation age directly promoted the *pqqC* gene abundance and AP content, whereas fertilization indirectly influenced P transformation by regulating microbial diversity. Our findings reveal that reclamation age and fertilization synergistically shape the inorganic P profile and the associated bacterial community, providing insights for developing targeted P management strategies in reclaimed lands.

## 1. Introduction

Coal is the primary energy source in China and is projected to maintain a share of approximately 50% in the nation’s primary energy consumption for the foreseeable future [1]. As a critical coal production base, Shanxi Province has supported economic development through extensive mining activities, which, however, have concurrently induced severe ecological issues, including land subsidence, vegetation loss, water and soil erosion, and desertification [2]. These disturbances lead to a significant degradation of ecosystem functions in mining areas. Land reclamation and ecological restoration are, therefore, recognized as vital measures for rehabilitating the local environment [3].

The newly constructed soil after mining disturbance, termed “minesoil,” is a fundamental component of the reclamation ecosystem, and its quality largely determines the success of restoration efforts [2]. Our previous studies have demonstrated that reclaimed minesoils in this region typically suffer from poor structure and nutrient deficiencies [4]. Furthermore, the calcareous nature of Shanxi soils causes strong phosphorus (P) fixation, resulting in low P bioavailability. This P limitation subsequently constrains nitrogen fixation efficiency [5], collectively impairing plant growth in these areas. Consequently, P availability has emerged as a major factor limiting the improvement of soil quality in reclaimed minesoils.

Soil microorganisms are the primary drivers of P cycling. They mediate the solubilization of insoluble P and the mineralization of organic P, thereby enhancing soil available P (AP) content, promoting plant growth, and serving as a key mechanism for sustainable agricultural development [6,7]. These microbial processes are largely governed by three categories of functional genes: those involved in inorganic P solubilization and organic P mineralization, P uptake and transport, and the P-starvation response regulation [8]. Specifically, inorganic P-solubilizing microorganisms can release organic acids to dissolve mineral P. Pyrroloquinoline quinone (PQQ), a cofactor for gluconic acid production, plays a central role in this solubilization process [9] and is regarded as one of the core functional genes in microbial P cycling [10,11]. Within the *pqq* gene cluster, *pqqC*, which encodes the enzyme catalyzing the final step of PQQ biosynthesis, is frequently used as a marker gene to trace and study inorganic P-solubilizing microbial communities. It is widely applied in phylogenetic and ecological analyses of these communities, linking their structure to P solubilization capacity [9,12]. Despite its importance, most research on P-solubilizing communities has focused on the alkaline phosphatase gene (*phoD*), while studies targeting *pqqC*-harboring microbial communities during mine soil reclamation remain scarce.

Agricultural practices, particularly fertilization, can significantly alter the diversity, composition, and function of soil microbial communities, thereby influencing microbially driven biogeochemical cycles. For instance, long-term nitrogen fertilization can reduce soil pH, potentially adversely affecting microbial P solubilization and mineralization capacities [8]. In contrast, another long-term (38 years) study revealed that nitrogen application fostered a more complex co-occurrence network and increased the abundance of key taxa within the *pqqC*-harboring bacterial community, ultimately enhancing soil P activation compared to treatments without N [13]. Under organic fertilization, manure application reshapes the *pqqC*-harboring bacterial community [12], significantly increasing the relative abundance of P-solubilizing genera like *Pseudomonas* and enhancing microbial network complexity [14], which collectively strengthens microbial P turnover. Moreover, organic fertilization elevates soil organic carbon (SOC) levels. Given that microbial P solubilization from apatite is often limited by organic carbon availability [15], and the *pqqC*-harboring community is itself regulated by SOC [16], this represents a key mechanism. In summary, functional genes like *pqqC* are sensitive to agricultural management and correlate with soil properties and functions [17]. However, findings are often inconsistent due to variations in fertilization regimes, climatic conditions, and soil properties such as pH. Consequently, the responses of P-solubilizing bacterial communities to fertilization and their underlying mechanisms are not fully understood, particularly in the context of mine soil reclamation. The ways in which fertilization influences these P-solubilizing communities and their associations with P fractions and soil physicochemical properties in reclaimed minesoils are still unclear.

To address this knowledge gap, we leveraged a long-term reclamation experiment in a Shanxi coal mining area to: (1) determine the effects of fertilization and reclamation age on inorganic P fractions; (2) characterize the response of the *pqqC*-harboring bacterial community to these factors; and (3) elucidate the interrelationships among soil properties, P fractions, and key microbial community features. We hypothesize that fertilization and increasing reclamation age will synergistically enhance P bioavailability by fostering a more specialized and efficient P-solubilizing microbial community. The findings from this study are expected to inform strategic management for improving soil health and accelerating ecological restoration in mining-affected landscapes.

## 2. Materials and Methods

### 2.1. Experimental Site and Design

The field experiment was conducted in Luojianggou Village, Wangqiao Town, Xiangyuan County, Shanxi Province, China (36°28′11.95′′ N, 113°00′52.57′′ E), within the mining field of the Wuyang Coal Mine, Lu’an Group. This region experiences a temperate continental monsoon climate with an average altitude of 980 m, a mean annual temperature of 9.5 °C, and a mean annual precipitation of 532.8 mm. The soil is classified as calcareous. Land subsidence due to coal mining commenced in the 1970s and stabilized around the year 2000. Reclamation initiatives began in the autumn of 2008, involving mechanical leveling of the subsided land followed by fertilization. The initial physicochemical properties of the reclaimed soil were as follows: pH 8.13, soil organic matter 7.58 g/kg, total nitrogen (TN) 0.49 g/kg, available P (AP) 2.49 mg/kg, and available potassium (AK) 72.6 mg/kg.

This study utilized plots with three different reclamation ages: 1, 5, and 10 years. Within each reclamation age, three fertilization treatments were established: no fertilizer (CK), chemical fertilizer (CF), and organic manure: (M). The experiment followed a randomized complete block design with three replications for each of the nine treatment combinations. Each plot covered an area of 100 m^2^. All fertilizers were applied annually as a basal dose prior to sowing. The application rates of the CF and M treatments were calculated to supply equivalent amounts of nitrogen (N), phosphorus (P), and potassium (K). The CF treatment consisted of urea (46% N), calcium superphosphate (16% P_2_O_5_), and potassium chloride (60% K_2_O). The M treatment consisted of fully composted chicken manure, containing 27.8% organic matter, 1.68% N, 1.54% P_2_O_5_, and 0.82% K_2_O. In line with local practices, maize was sown around May 1st and harvested around October 1st each year, with a planting density of 4000 plants per mu (approximately 667 m^2^). All plots received identical field management practices consistent with local conventional farming.

### 2.2. Soil Sampling and Chemical Analysis

Soil samples were collected from the 0–20 cm depth after the maize harvest using a five-point sampling method. A total of 27 samples were obtained (3 reclamation ages × 3 fertilization treatments × 3 replicates). After removing visible plant debris and stones, each sample was divided into two subsamples. One was immediately stored at −80 °C in a sterile bag for subsequent molecular DNA analysis. The other was air-dried, ground, and passed through appropriate sieves for physicochemical analyses.

Soil pH was directly measured using a pH meter (soil-to-water ratio of 1:2.5). Soil organic matter (SOM) was determined using the potassium dichromate oxidation method with external heating [18]. Total nitrogen (TN) was measured by the Kjeldahl method [19]. Total phosphorus (TP) and total potassium (TK) were determined after NaOH fusion by the molybdenum blue colorimetric method and flame photometry, respectively [20]. Available phosphorus (AP) was determined via the Olsen-P method, involving extraction with NaHCO_3_ (pH 8.5) followed by molybdenum-antimony anti-colorimetric analysis [21], while available potassium (AK) was extracted with 1 M ammonium acetate (NH_4_OAc) and analyzed by flame photometry [21]. Alkali-hydrolyzable nitrogen (AN) was determined by the alkaline diffusion method [22].

### 2.3. Analysis of Soil Inorganic Phosphorus Fractions

Soil inorganic P fractions were sequentially extracted according to a modified Hedley fractionation procedure [23,24,25]. Briefly, 0.5 g of soil (passed through a 100-mesh sieve) was sequentially extracted with deionized water, 0.5 M NaHCO_3_ (pH 8.2), 0.1 M NaOH, and 1 M HCl to obtain the following inorganic P (Pi) fractions: H_2_O-Pi, NaHCO_3_-Pi, NaOH-Pi, and HCl-Pi. The Pi concentration in each extract was quantified by the molybdenum blue colorimetric method. The remaining soil residue was then digested with concentrated H_2_SO_4_ and HClO_4_ to determine the residual P. Based on their bioavailability to plants and microorganisms, the P pools were categorized into three groups: (i) labile P (H_2_O-Pi and NaHCO_3_-Pi), (ii) moderately labile P (NaOH-Pi), and (iii) stable P (HCl-Pi and residual P) [26,27].

### 2.4. Soil DNA Extraction, pqqC Gene Quantification, and Sequencing

Total genomic DNA was extracted from 0.50 g of soil using the E.Z.N.A.^®^ Soil DNA Kit (Omega Bio-tek, Norcross, GA, USA) according to the manufacturer’s instructions. The DNA concentration and purity were assessed using a NanoDrop 2000 spectrophotometer (Thermo Scientific, Waltham, MA**,** USA).

The abundance of the *pqqC* gene was quantified by quantitative PCR (qPCR) on an ABI 7500 Real-Time PCR System (Applied Biosystems, Carlsbad, CA, USA). The reaction was performed using the primers *pqqC*-F (5′-AACCGCTTCTACTACCAG-3′) and *pqqC*-R (5′-GCGAACAGCTCGGTCAG-3′) [9]. This primer set is widely used in soil studies due to its broad specificity for key bacterial taxa involved in inorganic phosphate solubilization.

The 20 μL qPCR mixture 10 µL of 2× SYBR^®^ Premix (Biomed, Beijing, China),, 0.8 μL of each primer (10 μM), 1.4 μL of DNA template, and 7 μL of ddH_2_O. The thermal cycling conditions were: initial denaturation at 98 °C for 3 min; followed by 40 cycles of 98 °C for 20 s, 62 °C for 30 s, and 72 °C for 30 s. All samples were run in triplicate. A standard curve was generated using a 10-fold serial dilution of a plasmid carrying the target *pqqC* gene fragment (cloned into the pMD18-T vector), with the copy number calculated for each sample.

High-throughput sequencing of the *pqqC* gene was performed on an Illumina Nextseq 2000 platform by Meiji Bio (Shanghai, China). Raw sequencing reads were quality-filtered and trimmed using fastp (v0.19.6) and assembled using FLASH (v1.2.11). Operational Taxonomic Units (OTUs) were clustered at a 97% sequence identity threshold using UPARSE (v7.1). Taxonomic annotation of OTUs was performed by BLAST against the NT database (v20230830) with an 80% confidence threshold. The raw sequencing data generated in this study have been submitted to the NCBI Sequence Read Archive (SRA) with the accession number PRJNA1371913.

### 2.5. Statistical Analyses

A two-way analysis of variance (ANOVA) was performed using SPSS 25.0 (IBM, Armonk, NY, USA) to assess the effects of reclamation age, fertilization, and their interaction on soil properties, P fractions, and the relative abundance of key bacterial taxa. Data are presented as mean ± standard deviation, and differences were considered significant at *p* < 0.05. Pearson correlation analysis was used to examine relationships among soil variables. Principal coordinate analysis (PCoA) and redundancy analysis (RDA) were conducted using the “vegan” package in R (v2.5.3). Core microbial taxa were defined as those with an occurrence frequency >80%, and a co-occurrence network was constructed based on these core species. Pairwise correlations were calculated using the SparCC algorithm, and robust connections (absolute SparCC correlation >0.6 and *p* < 0.05) were retained for network construction. The topological properties of the network were visualized and calculated using Gephi (v0.9.2). A structural equation model (SEM) was developed using IBM SPSS AMOS 22 to elucidate the direct and indirect relationships among soil properties, P fractions, and the *pqqC*-harboring bacterial community.

## 3. Results

### 3.1. Soil Nutrients and Phosphorus Fractions in Reclaimed Soil

#### 3.1.1. Soil Nutrients

Reclamation duration and fertilization significantly influenced the basic nutrient status of the reclaimed soil (Table 1). Soil organic matter (SOM), total nitrogen (TN), and total phosphorus (TP) contents generally accumulated over the reclamation age. After 10 years, SOM content in the CK, CF, and M treatments increased by 54.9%, 56.1%, and 14.1%, respectively, compared to the first year. The M treatment significantly enhanced the contents of SOM, available phosphorus (AP), available nitrogen (AN), and TN. Notably, the M treatment consistently resulted in higher SOM levels than the CK and CF treatments across all reclamation ages, underscoring its role in building the soil carbon pool. Concurrently, the M treatment also maintained the highest AP content. Specifically, at 1, 5, and 10 years of reclamation, the AP content under M was 6.58, 1.43, and 1.62 times higher than that under CF, respectively, highlighting the superior P supply capacity of organic fertilizer. In contrast, the AP content under CF only showed a significant increase to 19.33 mg/kg at year 5 before declining, indicating potential P fixation and loss. Furthermore, both CF and M application reduced the pH of the calcareous soil, with a more pronounced decrease observed under the CF treatment.

Two-way ANOVA revealed a significant interactive effect of reclamation duration and fertilization on soil pH (*p* < 0.05), AP (*p* < 0.01), TN (*p* < 0.01), and total potassium (TK) (*p* < 0.01), demonstrating their joint regulation of soil nutrient dynamics.

#### 3.1.2. Distribution of Inorganic Phosphorus Fractions

**Figure 1:**
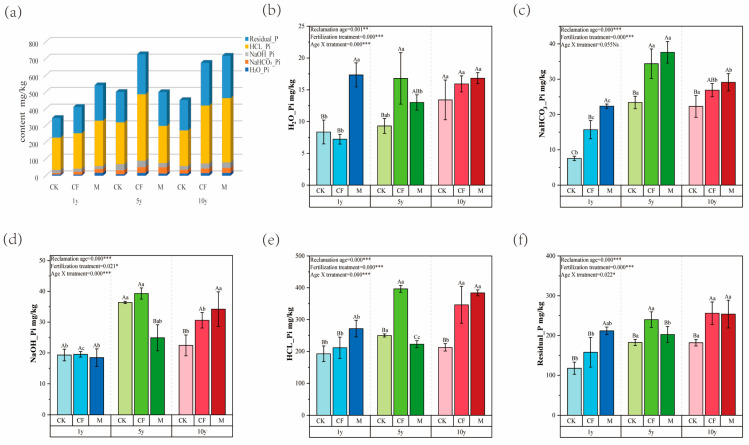
Changes in soil inorganic phosphorus (P) forms as influenced by fertilization and reclamation age. (**a**) Stacked bar chart showing the proportional distribution of sequential-extracted inorganic P fractions. (**b**–**f**) Bar charts depicting the concentrations of H_2_O-Pi (**b**), NaHCO_3_-Pi (**c**), NaOH-Pi (**d**), HCl-Pi (**e**), and Residual-P (**f**). Treatments: Control (CK), Chemical Fertilizer (CF), Organic Manure (M). Reclamation ages: 1 year (1y), 5 years (5y), and 10 years (10y). Values are means ± SD (n = 3). Statistical significance was evaluated by two-way ANOVA followed by Tukey’s HSD test: Lowercase letters (a, b, c) compare differences across reclamation ages within the same fertilization treatment. Uppercase letters (A, B, C) compare differences across fertilization treatments within the same reclamation age. ANOVA significance symbols: *** *p* < 0.001, ** *p* < 0.01, * *p* < 0.05.

Inorganic P distribution is depicted in Figure 1a, while Figure 1b–f resolve the response of each fraction to the treatments. Stable P fractions (HCl-Pi and Residual-P) dominated the inorganic phosphorus profile, collectively accounting for over 84.90% of the total. Among them, HCl-Pi was the predominant form, representing 44.45% to 55.72% (Figure 1a). The contents of NaHCO_3_-Pi, NaOH-Pi, HCl-Pi, and Residual-P increased significantly with increasing reclamation duration (Figure 1b–f), indicating a net enrichment of soil P through reclamation.

Fertilization differentially influenced the various P fractions. Except in the 5th year, the M treatment consistently resulted in higher levels of labile P (H_2_O-Pi and NaHCO_3_-Pi) compared to the CF and CK treatments across all ages (Figure 1b,c), suggesting a sustained supply of bioavailable P from organic inputs. The CF treatment primarily increased the contents of NaOH-Pi and HCl-Pi (Figure 1d,e). In contrast, these phosphorus fractions remained consistently low in the M treatment, particularly at the 5-year reclamation stage, where the levels in the M treatment corresponded to only 63.38% and 56.13% of those in the CF treatment, respectively.

The two-way ANOVA further confirmed that reclamation age, fertilization, and their interaction exerted highly significant effects (*p* < 0.001) on the contents of labile P (H_2_O-Pi), moderately labile P (NaOH-Pi), and stable P (HCl-Pi), underscoring their strong combined regulatory effect on soil inorganic P fractions.

#### 3.1.3. Relationships Between Soil Nutrients and Phosphorus Fractions

**Figure 2:**
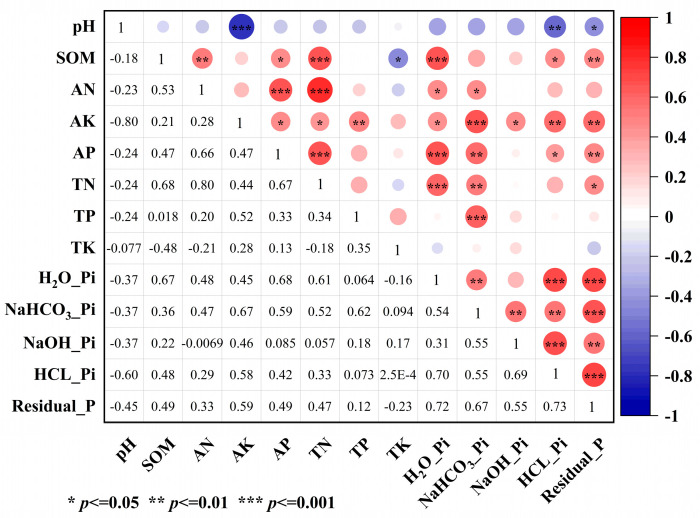
Pearson correlations between soil nutrients and inorganic phosphorus fractions. The lower-left section shows Pearson correlation coefficients (r), and the upper-right section indicates statistical significance (* *p* ≤ 0.05, ** *p* < 0.01, *** *p* < 0.001) using colored circles, where circle size and color represent the strength and direction of the correlation (red: positive, blue: negative, ranging from −1 to +1).

Pearson correlation analysis revealed close associations between soil physicochemical properties and P fractions (Figure 2). SOM showed significant positive correlations with H_2_O-Pi (r = 0.67, *p* < 0.001) and Residual-P (r = 0.49, *p* < 0.01). Soil AP and TN were also significantly positively correlated with H_2_O-Pi (r = 0.68, *p* < 0.001) and NaHCO_3_-Pi (r = 0.54, *p* < 0.01).

Significant correlations were also observed among different P fractions. For instance, H_2_O-Pi was not only correlated with NaHCO_3_-Pi (r = 0.54, *p* < 0.01) but also showed significant positive correlations with stable P fractions (HCl-Pi and Residual-P; *p* < 0.001). NaHCO_3_-Pi was also positively correlated with moderately labile P (NaOH-Pi) and stable P (HCl-Pi, Residual-P). These correlations suggest potential transformations between different P pools, which are influenced by soil physicochemical properties.

### 3.2. pqqC Gene Abundance and α-Diversity

**Figure 3:**
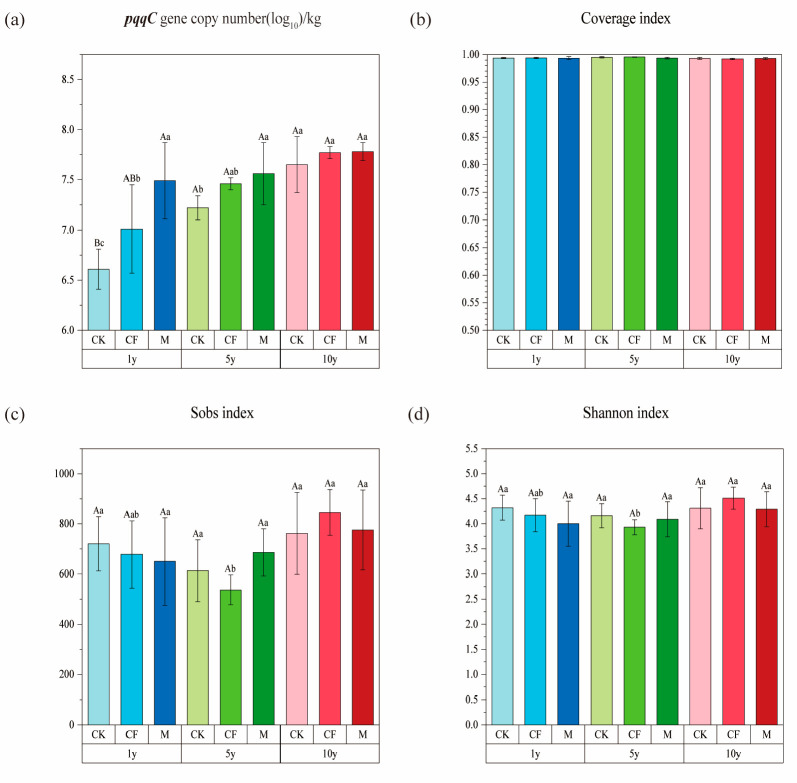
*pqqC* gene abundance and α-diversity indices in reclaimed soils under different fertilizations and reclamation ages. (**a**) *pqqC* Gene abundance. (**b**) Coverage index. (**c**) Observed species (Sobs index). (**d**) Shannon diversity index. Fertilization treatments: CK (control), CF (chemical fertilizer), M (organic manure). Reclamation ages: 1 year (1y), 5 years (5y), and 10 years (10y). Bars represent means ± standard deviation (n = 3). Statistical significance was evaluated by two-way ANOVA followed by Tukey’s HSD test: Lowercase letters (a, b, c) compare differences across reclamation ages within the same fertilization treatment. Uppercase letters (A, B) compare differences across fertilization treatments within the same reclamation age.

Fertilization significantly affected the abundance of the *pqqC* gene in the soil (Figure 3a). Within the same reclamation age, the *pqqC* gene abundance consistently followed the order: M > CF > CK. This indicates that fertilization, particularly organic amendment, enhanced the abundance of P-solubilizing microorganisms harboring the *pqqC* gene.

The coverage of *pqqC* primers (Figure 3b), all samples covered more than 99%, indicating that sequencing depth can ensure that the vast majority of species are measured. In the early reclamation stages (1 and 5 years), the Sobs and Shannon indices of the M and CF treatments were lower than those of the CK treatment (Figure 3c,d). This effect was particularly significant for the CF treatment (*p* < 0.05), suggesting that short-term fertilization initially disturbed the *pqqC*-harboring microbial community. By year 10, the diversity indices across all treatments reached their peak. The Sobs and Shannon indices in the CF and M treatments were comparable to or even higher than those in CK, indicating that long-term reclamation facilitated the recovery and stabilization of microbial diversity.

### 3.3. Composition of the pqqC-Harboring Bacterial Community

**Figure 4:**
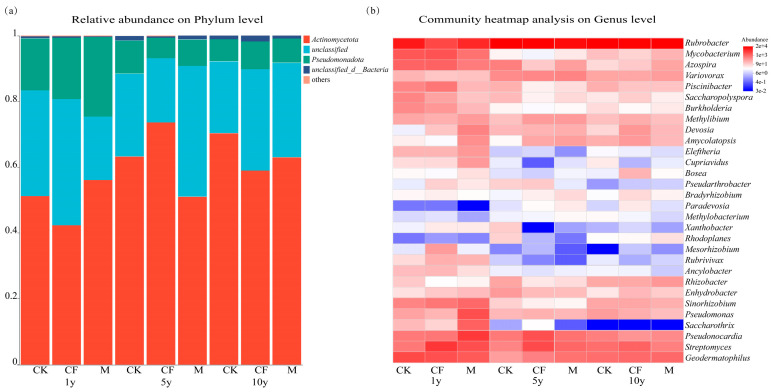
Microbial community structure of *pqqC*-harboring bacteria. (**a**) Phylum-level composition. (**b**) Heatmap of the top 30 genera (Z-score normalized). Treatments: Control (CK), Chemical Fertilizer (CF), Organic Manure (M). Reclamation ages: 1 year (1y), 5 years (5y), and 10 years (10y). In the heatmap, red and blue shades denote higher and lower relative abundance, respectively.

At the phylum level, *Actinobacteria* (relative abundance 42.41–73.62%) and *Pseudomonadota* (relative abundance 6.31–24.10%) were the dominant phyla across all samples (Figure 4a). A heatmap displayed the abundance variations of the top 30 genera (Figure 4b). Predominant genera included *Rubrobacter* (1.49–9.42%), *Streptomyces* (0.26–2.05%), *Pseudonocardia* (0.21–1.98%), *Pseudomonas* (0.07–1.22%), and *Azospira* (0.03–0.76%). The relative abundance of *Rubrobacter* increased with reclamation duration in all treatments. After 10 years, its abundance in CK, CF, and M treatments was 2.14, 4.98, and 2.79 times higher, respectively, than at year 1. In contrast, the relative abundance of *Pseudonocardia* decreased over time, showing reductions of 59.30%, 46.80%, and 32.62% in the CK, CF, and M treatments at year 10 compared to year 1. The relative abundance of *Streptomyces* exhibited contrasting responses to fertilization: it increased by 23.03% in CK after 10 years but decreased by 64.21% and 70.56% in the CF and M treatments, respectively.

### 3.4. Microbial Community Structure and Environmental Drivers

**Figure 5:**
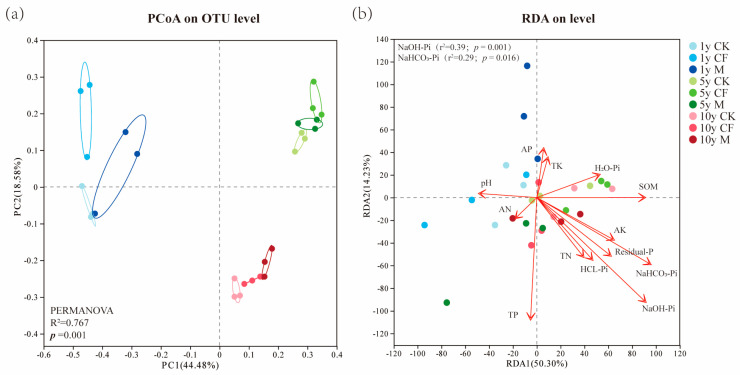
Multivariate analysis of the *pqqC*-harboring microbial community structure. (**a**) Principal Coordinates Analysis (PCoA) based on Bray–Curtis distances (**b**) Redundancy Analysis (RDA) displaying the relationship between microbial community composition and key environmental variables. Samples are colored by fertilization treatment [Control (CK), Chemical Fertilizer (CF), Organic Manure (M)] and reclamation ages: 1 year (1y), 5 years (5y), and 10 years (10y).

Principal coordinate analysis (PCoA) based on Bray–Curtis distances revealed a clear separation of the *pqqC*-harboring microbial communities by reclamation age, forming three distinct clusters (Figure 5a). This indicates that reclamation age was the primary factor shaping community structure, while fertilization had a secondary effect.

Redundancy analysis (RDA) illustrated the influence of soil properties and P fractions on community composition (Figure 5b). The first RDA axis (RDA1) alone explained 50.3% of the community variation, and the two axes together explained 64.5%, indicating that the selected environmental variables effectively explained the community differentiation. NaOH-Pi (r^2^ = 0.39, *p* = 0.001) and NaHCO_3_-Pi (r^2^ = 0.29, *p* = 0.016) (Table S2), were identified as the key environmental factors driving the structural divergence of the *pqqC*-harboring community.

### 3.5. Co-Occurrence Network Analysis of Core Taxa

**Figure 6:**
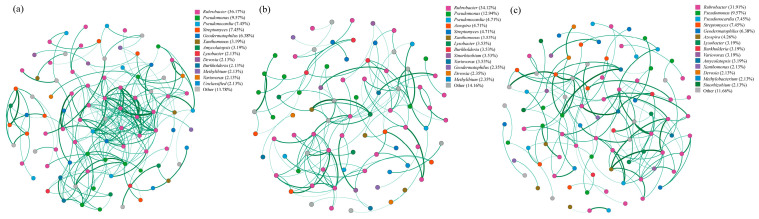
Co-occurrence networks of the core *pqqC*-harboring microbial taxa at different reclamation ages: (**a**) 1 year, (**b**) 5 years, and (**c**) 10 years. Networks were constructed based on persistent OTUs (occurrence frequency > 80%) using SparCC correlations. Only robust interactions (|r| > 0.6, *p* < 0.05) are displayed. Nodes represent OTUs (colored by genus), and edges (green lines) depict positive correlations, whose visual weight is linearly scaled to the correlation strength. The network layout was generated using a force-directed algorithm in Gephi.

Based on occurrence frequency, microbial taxa were categorized as transient (<20%), intermediate (20–80%), or persistent (>80%) species. Persistent species considered the core community (Table S3), were used to construct co-occurrence networks (Figure 6).

Analysis of network topology parameters (Table S4) showed that the network at year 1 (Figure 6a) had the highest connectivity, indicating complex inter-taxa relationships. The key taxa in the co-occurrence network included *Rubrobacter* (36.17%), *Pseudomonas* (9.57%), *Pseudonocardia* (7.45%), and *Streptomyces* (7.45%). By year 5 (Figure 6b), network nodes and aggregation coefficient decreased by 9.57% and 8.12% compared to the year 1. The dominant taxa shifted to *Rubrobacter* (34.12%), *Pseudomonas* (12.94%), and *Pseudonocardia* (4.71%), reflecting a reorganization of microbial interactions. At year 10 (Figure 6c), the network structure simplified further, with aggregation coefficient declining by 6.82%, respectively, relative to the year 5. The network was primarily governed by *Rubrobacter* (31.91%), *Pseudomonas* (9.57%), and *Pseudonocardia* (7.45%).

### 3.6. Structural Equation Modeling (SEM)

**Figure 7:**
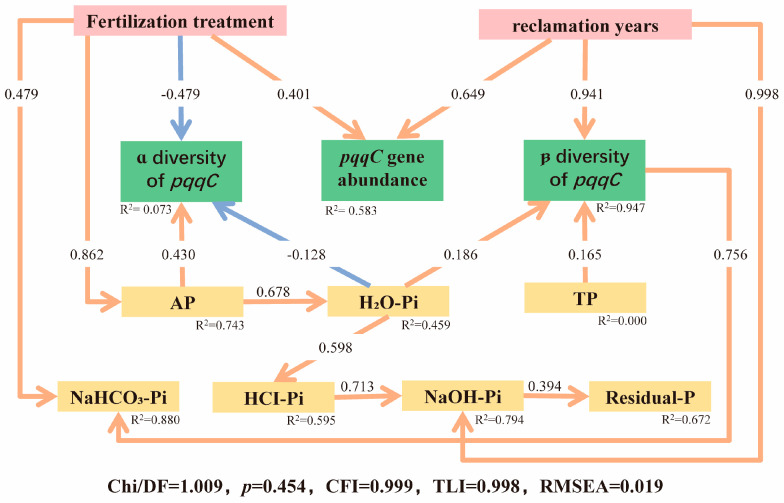
Structural equation model (SEM) testing the direct and indirect effects of reclamation age and fertilization on soil phosphorus availability and the *pqqC*-harboring bacterial community. Arrows represent hypothesized causal pathways. Solid red arrows indicate significant positive effects, while solid blue arrows indicate significant negative effects. Non-significant paths are omitted for clarity. Path coefficients are standardized, and R^2^ values represent the proportion of variance explained for each endogenous variable.

Structural equation modeling (SEM) (Figure 7) clearly revealed the direct and indirect effects of fertilization treatment and reclamation age on soil phosphorus forms and the *pqqC*-harboring microbial community. Reclamation age exhibited strong direct positive effects on *pqqC* gene abundance (path coefficient = 0.649; R^2^ = 0.583), microbial community β-diversity (path coefficient = 0.941; R^2^ = 0.947), and NaOH-Pi (path coefficient = 0.998; R^2^ = 0.794). Community β-diversity, in turn, directly and positively influenced NaHCO_3_-Pi (path coefficient = 0.756).

In contrast, the influence of fertilization treatment involved more complex pathways. While it exerted a direct negative effect on the α-diversity of the *pqqC* microbial community (path coefficient = −0.479), it significantly increased AP (path coefficient = 0.862) and NaHCO_3_-Pi (path coefficient = 0.479) contents. AP served as a central hub in the network (R^2^ = 0.743), directly and positively affecting the α-diversity of the *pqqC* community (path coefficient = 0.430) and strongly driving the formation of H_2_O-Pi (path coefficient = 0.678). Subsequently, H_2_O-Pi negatively influenced the α-diversity (path coefficient = −0.128) and positively influenced the β-diversity (path coefficient = 0.186) of the *pqqC* community.

## 4. Discussion

### 4.1. Influence of Reclamation Age and Fertilization on the Soil Phosphorus Pool and Its Availability

Low available phosphorus (AP) is a primary constraint on soil fertility and crop yield, particularly in calcareous soils [28]. Fertilization, as a common agricultural practice to enhance P availability [17], is often employed to rapidly improve the nutrient status of reclaimed soils. Our study focused on a calcareous reclaimed soil in a Shanxi coal mining area, where soil nutrients and AP were inherently low due to mining-induced degradation and the region’s calcareous nature [4]. We found that long-term fertilization significantly reshaped the soil nutrient environment and P fraction distribution, with organic fertilizer (M) exhibiting the most profound impact (Table 1).

Consistent with numerous studies [29,30,31,32,33,34], the M treatment significantly increased soil SOM, AP, and TN compared to the CF treatment. This underscores the superiority of organic amendments over mineral fertilizers in enhancing soil fertility. The categorization of soil P into labile, moderately labile, and stable pools based on bioavailability is crucial for understanding P dynamics [17,35]. Our results revealed that the M treatment led to higher contents of labile P (H_2_O-Pi and NaHCO_3_-Pi) but lower contents of moderately labile P (NaOH-Pi) compared to the CF treatment (Figure 1). This indicates that organic fertilization not only supplements soil nutrients but, more importantly, promotes the activation of moderately labile P into more readily available forms.

The strong positive correlation between SOM and H_2_O-Pi (*p* < 0.001, Figure 2) suggests a potential mechanism. Organic inputs likely elevate SOM, which can alleviate microbial P limitation by providing labile carbon. Simultaneously, they improve soil aggregate structure, creating a more favorable habitat and supplying energy sources for microorganisms [36,37,38]. These improvements can stimulate microbially driven P cycling, thereby enhancing P availability [39], a process intimately linked to the function of *pqqC*-harboring microbes [40,41,42]. This pattern was also reflected in the SEM (Figure 7), wherein both reclamation and fertilization promoted the recovery of *pqqC* gene abundance and community β-diversity, which in turn exerted a direct positive effect on the labile phosphorus pool (NaHCO_3_-Pi).

In contrast, a large proportion (nearly 80%) of applied chemical P fertilizer is often fixed in the soil and becomes unavailable to plants [43]. Our findings align with this, showing that CF application primarily enriched the moderately labile (NaOH-Pi) and stable (HCl-Pi) P pools, especially after 5 years of consecutive application (Figure 1d,e), demonstrating significant P fixation [44]. HCl-Pi, identified as the dominant inorganic P fraction in these soils, primarily consists of Ca-bound P (Ca-P) as per the Hedley fractionation scheme [24], which is consistent with the calcareous nature of the mine soils. Therefore, from the perspective of both nutrient accumulation and sustainable P supply in these calcareous reclaimed soils, organic fertilizer demonstrates clear advantages over chemical fertilizer.

### 4.2. Effects of Reclamation Age and Fertilization on the pqqC Gene Abundance and Microbial Community

Phosphorus availability significantly influences soil microbial dynamics; deficiency can suppress microbial growth, while P addition often increases microbial biomass [8]. Our qPCR results demonstrated that both CF and M application enhanced *pqqC* gene abundance, which increased progressively with reclamation age (Figure 3a). Notably, the M treatment consistently supported a higher *pqqC* abundance than the CF treatment at all time points, indicating its superior capacity for enriching P-solubilizing microorganisms, regardless of the application period [37].

The response of community α-diversity to fertilization was stage-dependent. In early reclamation stages (≤5 years), both CF and M treatments resulted in lower Sobs and Shannon indices compared to the unfertilized control (CK) (Figure 3c,d). In P-deficient reclaimed soils, the sudden input of available P from fertilization likely provides a strong selective pressure, stimulating the proliferation of specific, highly efficient P-solubilizing taxa (increasing gene abundance) while reducing overall taxonomic diversity. This “specialization” effect is corroborated by our structural equation model (SEM, Figure 7), which showed a positive direct effect of fertilization on AP and *pqqC* abundance but a negative direct effect on α-diversity. Similar findings have been reported elsewhere, where increased soil AP led to reduced diversity in P-solubilizing microbial communities, suggesting environmental filtering. Long-term reclamation (10 years) appeared to facilitate the recovery and stabilization of *pqqC*-harboring community diversity.

The microbial co-occurrence network (Figure 6) analysis revealed that the number of connections was highest in the year 1, suggesting that both reclamation and fertilization enhanced interspecific interactions [45,46]. Similar findings have been reported in other studies, where external nutrient inputs were shown to strengthen correlations among microbial taxa [47,48]. Interestingly, as the reclamation progressed, the number of network connections gradually decreased at 5 and 10 years (Table S3), indicating a significant decline in interspecific interactions over time. This observation aligns with community succession theory, which posits that soil bacterial communities transition from an initial phase of rapid response and intense competitive selection toward a mature and stable stage [49]. Consistently, Guseva [50] and Hu [51] also reported a shift toward a “functionally stable state” in microbial networks after five years of restoration. The clear separation of communities by reclamation duration in the PCoA (Figure 5a) reinforces that temporal succession, rather than fertilization, is the overarching driver of microbial community assembly in these reclaimed ecosystems. This progression aligns with the natural restoration paradigm of coordinated “soil development-microbial community-function” evolution.

In this study, the dominant bacterial phyla harboring the *pqqC* gene were *Pseudomonadota* and *Actinobacteria*, which is consistent with observations in other agricultural systems [8,9,17]. Notably, the key taxa functioning as hubs in the microbial co-occurrence network (Figure 5)—*Pseudomonas, Rubrobacter, Pseudonocardia*, and *Streptomyces*—belong to these two phyla. This further indicates that *Pseudomonadota* and *Actinobacteria* serve as critical carriers of the *pqqC* gene and represent important phosphorus-solubilizing microorganisms [8,9]. *Pseudomonas*, a well-known P-solubilizer through organic acid secretion [52], constituted 9.57–12.94% of the network and likely acts as a primary executor of P dissolution. *Streptomyces*, another genus with P-solubilizing capacity, also plays a critical role as an antibiotic producer, potentially mitigating competition from harmful microorganisms and fostering a favorable niche for other solubilizers like *Pseudomonas*. Its established role as a key predictor of AP in maize rhizospheres [53] supports its functional importance in our system. However, its abundance decreased after 5 and 10 years of reclamation, probably because *Streptomyces*, as an oligotrophic microorganism (preferring low concentrations of organic nutrients), is less competitive than copiotrophic bacteria (such as some taxa of *Pseudomonadota*) in the high-nutrient environment brought by fertilization [54]. In addition, the decrease in soil pH caused by fertilization (Table 1) can also inhibit its growth and reproduction [55]. *Rubrobacter* is not a common phosphorus-dissolving bacterium, but it has also been reported as a phosphorus-dissolving bacterium in similar studies on the diversity of the *pqqC* gene [56,57,58]. It exhibits characteristics of drought and barren tolerance [59], and studies have found that in ecosystems under long-term drought stress, the relative abundance of *Rubrobacter* is significantly correlated with soil organic carbon [60].Our results also found that seven high-abundance OTUs within the genus *Rubrobacter* were significantly correlated with soil SOM, as well as NaHCO_3_-Pi and NaOH-Pi (Figure S1). It is speculated that this group can not only solubilize phosphorus in arid environments but also play an important role in organic carbon transformation. Its metabolic products may provide a carbon source for other phosphorus-solubilizing bacteria, forming a metabolic chain of “organic matter degradation—carbon source supply—phosphorus dissolution synergy.”

These findings collectively suggest that P solubilization is not a ubiquitous function across the microbial community but is primarily driven by a consortium of key taxa, including *Pseudomonas*, *Pseudonocardia*, *Streptomyces*, and *Rubrobacter*. The shifting dominance and interactions among these taxa illustrate a successional trajectory of the P-solubilizing network—from an initial, complex collaborative assemblage towards a later, more streamlined and modular functional structure. This underscores that successful soil reclamation involves not only revegetation but also the crucial restoration and optimization of the subsurface microbial “workforce.” Monitoring the dynamics of these key microbial consortia could therefore serve as a valuable bio-indicator for assessing the efficacy and trajectory of ecological restoration in reclaimed lands.

## 5. Conclusions

This study systematically elucidates the synergistic effects of fertilization and reclamation age on inorganic phosphorus (P) transformation and the *pqqC*-harboring bacterial community in reclaimed minesoils. We conclude that: Organic fertilizer is paramount for enhancing P bioavailability in calcareous reclaimed soils, primarily by increasing labile P pools and counteracting P fixation. Reclamation age is the dominant driver of microbial community succession, directly boosting the abundance and shaping the structure of *pqqC*-harboring bacteria, leading to more complex and stable networks. Fertilization acts indirectly, primarily by modulating microbial diversity which in turn influences P transformations, with organic inputs fostering a more proficient P-solubilizing consortium. The interaction is synergistic: Long-term reclamation provides the template for community development, which organic fertilization then effectively “manages” to optimize P cycling. By integrating P fractionation, microbial community analysis, and network modeling, we move beyond correlation to reveal the mechanistic interplay between management, microbes, and soil function. These insights advocate for the combined use of organic amendments and natural succession in developing sustainable P management strategies for the restoration of degraded mining ecosystems.

## Figures and Tables

**Table 1:** Basic soil nutrient properties across fertilization treatments and reclamation ages.

Reclamation Age	Fertilization Treatment	pH	SOM (g/kg)	AN (mg/kg)	AK (mg/kg)	AP (mg/kg)	TN (g/kg)	TP (g/kg)	TK (g/kg)
1	CK	8.07 ± 0.05 Aa	7.71 ± 0.94 Ab	23.89 ± 2.36 Aab	76.68 ± 4.00 Ac	1.58 ± 0.41 Bb	0.53 ± 0.14 Ab	0.19 ± 0.08 Aa	14.27 ± 0.22 Aa
	CF	7.91 ± 0.04 Ba	7.88 ± 1.31 Ac	24.57 ± 3.55 Ab	164.73 ± 88.32 Ab	4.92 ± 0.49 Bc	0.42 ± 0.15 Ab	0.20 ± 0.01 Aa	13.97 ± 0.30 ABa
	M	8.01 ± 0.05 Aa	10.98 ± 3.23 Aa	30.71 ± 12.79 Aa	139.38 ± 53.30 Aa	32.36 ± 9.20 Aa	0.73 ± 0.38 Aa	0.19 ± 0.17 Ab	13.63 ± 0.34 Bab
5	CK	8.11 ± 0.05 Aa	8.38 ± 0.07 Ab	16.38 ± 2.05 Cb	104.70 ± 4.00 Cb	2.04 ± 0.89 Bb	0.17 ± 0.05 Cc	0.25 ± 0.07 Ba	13.93 ± 0.27 Aa
	CF	7.73 ± 0.11 Bb	9.64 ± 0.59 Ab	26.62 ± 2.05 Bb	288.80 ± 4.00 Aa	19.33 ± 3.18 Aa	0.71 ± 0.11 Ba	0.55 ± 0.27 ABa	15.31 ± 1.37 Aa
	M	7.99 ± 0.08 Aa	9.76 ± 1.38 Aa	40.27 ± 6.58 Aa	195.42 ± 8.33 Ba	27.45 ± 6.63 Aa	1.10 ± 0.10 Aa	0.69 ± 0.13 Aa	14.14 ± 0.45 Aa
10	CK	8.03 ± 0.03 Aa	11.94 ± 1.26 Aa	27.98 ± 8.52 Aa	126.04 ± 8.33 Ca	4.13 ± 0.63 Ba	0.79 ± 0.04 Aa	0.24 ± 0.06 Aa	12.25 ± 0.20 Bb
	CF	7.82 ± 0.07 Bab	12.30 ± 0.35 Aa	33.44 ± 3.13 Aa	194.08 ± 14.06 Aab	13.37 ± 1.78 ABb	0.88 ± 0.03 Aa	0.32 ± 0.29 Aa	11.99 ± 0.20 Bb
	M	8.01 ± 0.03 Aa	12.53 ± 0.81 Aa	37.54 ± 5.15 Aa	159.40 ± 22.76 Ba	21.69 ± 7.96 Aa	1.00 ± 0.21 Aa	0.14 ± 0.18 Ab	13.07 ± 0.15 Ab
Two-way ANOVA (*p* values)									
Reclamation age		0.140 Ns	0.000 ***	0.083 Ns	0.003 **	0.274 Ns	0.002 **	0.002 **	0.000 ***
Fertilization treatment		0.000 ***	0.048 *	0.001 **	0.000 ***	0.000 ***	0.000 ***	0.235 Ns	0.557 Ns
Age X treatment		0.038 *	0.309 Ns	0.186 Ns	0.093 Ns	0.009 **	0.005 **	0.091 Ns	0.007 **

Data represent mean ± SD (n = 3). Statistical significance was evaluated by two-way ANOVA followed by Tukey’s HSD test: Lowercase letters (a, b, c) compare differences across reclamation ages within the same fertilization treatment. Uppercase letters (A, B, C) compare differences across fertilization treatments within the same reclamation age. ANOVA significance symbols: *** *p* < 0.001, ** *p* < 0.01, * *p* < 0.05, Ns *p* ≥ 0.05. Statistical parameters from the two-way ANOVA, including the F-value, degrees of freedom (df), mean square (MS), sum of squares (SS), and *p*-value, are provided in Supplementary Table S1.

## Data Availability

The original contributions presented in this study are included in the article/Supplementary Material. Further inquiries can be directed to the corresponding author.

## Supplementary Materials

The following supporting information can be downloaded at: https://www.mdpi.com/article/10.3390/microorganisms13122855/s1, Figure S1: Pearson correlations between high-abundance OTUs of the genus *Rubrobacter* and soil nutrients/phosphorus fractions; Table S1: Statistical parameters from the two-way ANOVA; Table S2: The individual contribution results of each environmental factor; Table S3: Persistent OTUs identified as the core microbial community; Table S4: Topological properties of the microbial co-occurrence networks across reclamation chronosequence.
