# Supplementary figures and images for: Synergistic Effects of Fertilization and Reclamation Age on Inorganic Phosphorus Fractions and the pqqC-Harboring Bacterial Community in Reclaimed Coal Mining Soils
Source: Microorganisms. 2025 Dec 16;13(12):2855. doi: 10.3390/microorganisms13122855 (PMC12735894; doi:10.3390/microorganisms13122855)

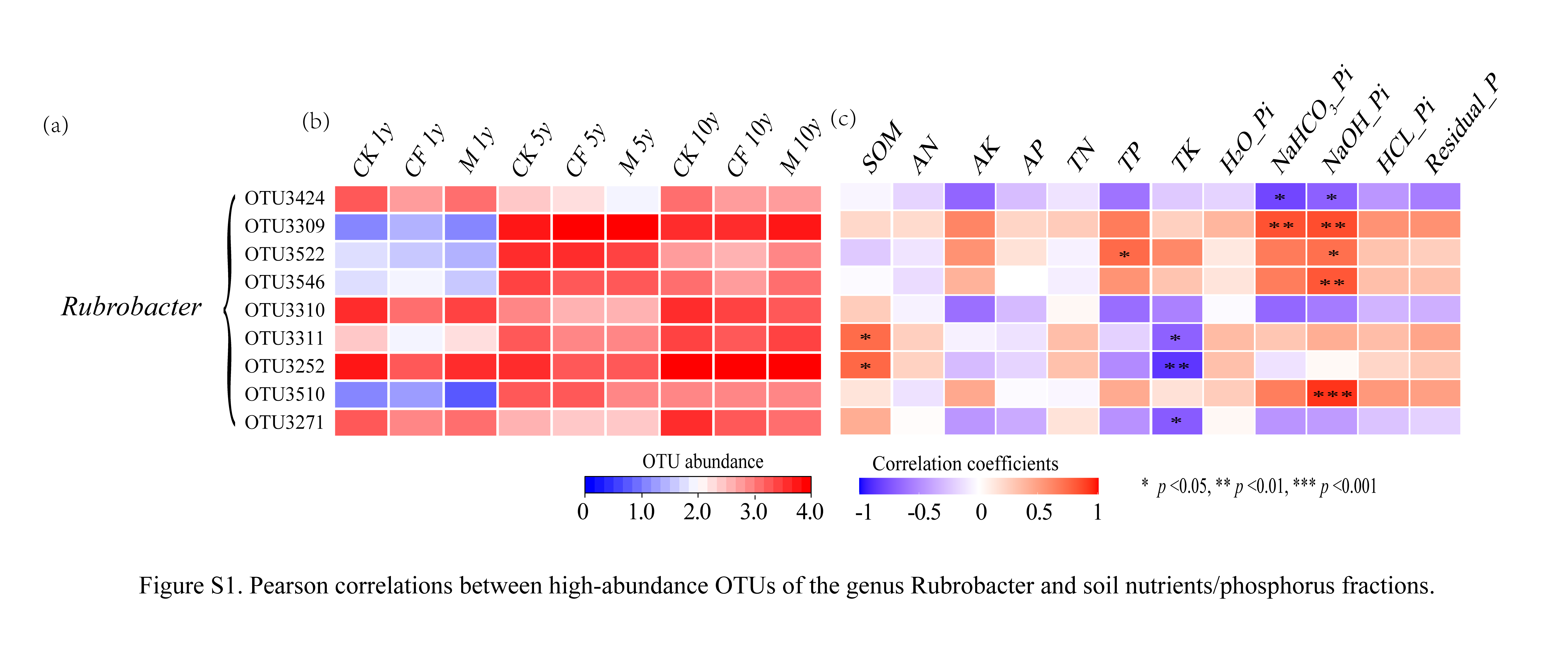

Supplement: Supplementary file 1 [file microorganisms-13-02855-s001.zip › Figure S1.tif]
